# Argumentative style of parent-child interactions: A case study

**DOI:** 10.1371/journal.pone.0318310

**Published:** 2025-03-18

**Authors:** Xian-Ju Yang, Chun-Chun Shen-Tu

**Affiliations:** School of Foreign Languages, Zhejiang Gongshang University Hangzhou College of Commerce, Hangzhou, Zhejiang, China; Simon Fraser University, CANADA

## Abstract

Parent-child argumentation is a unique form of communication, as it combines persuasion, emotional exchange, and instructional dynamics shaped by contextual factors and cultural norms. To fully understand how a parent balances dialectical reasonableness with rhetorical adaptability to resolve conflicts and foster cooperation with the child within specific context, this study investigates the argumentative style of parent-child interaction within the Chinese context, focusing on the interplay of cultural values, educational goals, and argumentative practices in family interaction. Within a corpus of 20 hours of recordings of parent-child conversations concerning educational topics from 5 Chinese families, a conversation between a mother and a son during homework was selected and qualitatively analyzed, based on the framework of Argumentative Style developed from the standard model of pragma-dialectics. The findings highlight the predominant use of an engaged argumentative style in the case, which features the parent’s radiating commitment in topical selection, communality in adaptation to audience demand and inclusiveness in presentational devices. An occasional shift to a detached style was also identified, particularly when the authority figure of the teacher was invoked. The subtle balance between nurturing parental involvement and reinforcing respect for established norms is reflective of the broad Chinese cultural values that parents bear significant responsibilities for children’s academic success, and act as guides and enforcers in family education. By investigating the roles different argumentative styles play in real-life parent-child interaction, this study provides implications for developing effective communication strategies in family education and highlights the significance of culturally informed argumentative practices.

## 1. Introduction

Parent-child argumentation, as a significant component of daily family communication, plays a pivotal role in the development of children’s cognitive abilities, language acquisition, and socialization [1]. Unlike the communicative activity types in such domains as law, politics and academics, parent-child argumentation is not constrained by institutional settings and conventionalized expressions. However, due to the particular roles parents and children play in family life, the argumentation is characterized by a certain pattern, i.e., “on the one hand, the parents try to convince their children to accept their rules and prescriptions, on the other, the children cast doubt on the parents’ standpoint and can ask their parents to make the reasons on which their standpoint is based more explicit” [2, p.44]. Meanwhile, the argumentative strategies adopted by parents are characterized by cultural and regional differences, as was suggested by Arcidiacono and Bova’s research on Italian and Swiss families [3, 4]. Therefore, parent-child argumentation can be seen as a type of communicative activity influenced by the macro context and its features need to be explored within particular contexts.

### 1.1. Parent-child argumentation

In recent years, studies on parent-child argumentation have gradually gained academic attention, particularly those conducted within the framework of pragma-dialectics. A number of studies [2–7] focusing on parent-child argumentation at mealtimes identified some typical argumentative strategies adopted by parents to resolve differences of opinion with their children, including ironic comments, appeal to the amount and quality of food, appeal to consistency, arguments from authority, arguments from analogy, etc. These studies primarily summarized typical argumentation strategies in parental discourse based on extensive research data, which means that the pragma-dialectical model only served as the criteria for the reconstruction of parent-child argumentative discussions and the identification of the arguments put forth by parents in particular discussion stages. Furthermore, Huang and Ye [8] conducted a case study to examine the argument schemes used by a Chinese parent to convince her daughter to “take the baby seat” in a car, finding that the mother’s arguments violated the rules for critical discussion and gave rise to logical fallacies.

Other relevant studies adopted a multi-disciplinary approach to explore the relationship between parent-child interactions and children’s socialization development. For instance, studies combining sociocultural perspectives with the pragma-dialectic framework revealed that children’s reasoning patterns and abilities were often acquired from adults’ argumentation and influenced by social norms and cultural contexts [9–11]. Additionally, some studies drew on Conversation Analysis informed approach to examine specific stages of parent-child interactions such as parents’ initial inquiries about homework [12] and parents’ response to children’s requests for alternative activities at mealtimes [13], illustrating how conversational practices were utilized by parents to manage specific situations, such as handling requests during mealtimes or initiating discussions about homework.

Beyond the conversational characteristics of parent-child interactions, some broader issues implied in these interactions have been equally explored. For example, Caronia and Colla [14] examined how parents referenced and exploited the material agency (e.g., pens, pencil cases, books) in homework-related exchanges, suggesting that object-mediated interactions underpinned the activity of homework tutoring and embodied school culture in family education. Similarly, another study by Caronia and Colla [15] illustrated another dimension beyond the academic focus of homework discussions: the “ethical affordances” that trigger moral talk, through which children are socialized into moral beings.

In summary, existing research has primarily focused on the argumentative strategies, reasoning patterns, and cultural and moral socialization of parent-child interactions. While the pragma-dialectical framework holds considerable methodological significance, much of the research reconstructed and interpreted the interactions based on its ideal model without systematically examining the rhetorical effectiveness of argumentation associated with specific contextual factors. In this regard, the sociocultural perspectives have offered meaningful insights into how the specifics of context influence the choice and effectiveness of the conversational strategies involved in parent-child interactions, but they are insufficient in evaluating the rationality of real-life argumentation. As such, the present study draws on the newly developed notion of Argumentative Style in pragma-dialectics which offers a robust framework for understanding not only the logical structure of arguments but also the real-life dynamics and contextual factors that shape the ways various argumentative moves are made, received, and negotiated in the unique setting of parent-child interactions.

### 1.2. Parental involvement in homework

The cultural emphasis on academic success in China motivates Chinese parents to be actively involved in children’s education, particularly in homework tutoring. Parental involvement in homework within the Chinese context has been explored as an intersection where academic, emotional, and cultural exchanges occur, influencing not only children’s development but also the broader family dynamics [16–20].

Survey results indicated that approximately 74% of Chinese elementary school parents were frequently involved in their children’s homework, with around 40% providing daily tutoring [19]. During the process, parents usually accompanied, supervised, urged, or assisted their children, but might also impose pressure and give brusque orders due to educational anxiety. In response, children raised questions, expressed objections, or even displayed resistant emotions [17]. This argumentative nature of parent-child interactions during homework tutoring enables observation of the communicative practices, cultural influences, and argumentative strategies that shape parental discourse, providing insights into the consistent ways in which parents resolve differences of opinion with their children in specific contexts.

## 2. Theoretical framework

This study adopts the theoretical framework of Argumentative Style developed by van Eemeren [21–23]. Argumentative Style refers to:

*The particular shape systematically and consistently given to the selection of topical choices, adaptation to audience demand, and exploitation of presentational devices in the strategic maneuvering in a representative part of an argumentative discourse, which manifests itself in the argumentative discourse in the argumentative moves included in the analytic overview, the dialectical routes indicated by the argumentative pattern, and the strategic considerations reflected in the strategic design* [23, p.10].

According to this definition, Argumentative Style covers three dimensions: 1) the selection of topical choices manifested in the selection of such argumentative moves as the standpoint, point of departure and arguments, etc.; 2) the adaptation to audience demand realized by various argumentative moves; 3) the exploitation of presentational devices such as verbal or non-verbal means to push forward argumentative moves. To explore the properties of the three dimensions, we need to first reconstruct the argumentative discourse by identifying those analytically relevant moves to shape an “analytical overview” and then discern the argumentative pattern to clarify the “dialectical routes”, and finally analyze the strategic considerations involved in the argumentative process to present the “strategic design” [22]. The three steps constitute what we call “analysis of argumentative discourse” which will be elaborated on in the following section.

### 2.1. Analysis of the argumentative discourse

The first step of argumentative discourse analysis is shaping an “analytic overview” by reconstructing the argumentative discourse and identifying those analytically relevant moves. According to the ideal model of pragma-dialectics, four stages can be distinguished in an argumentative discussion. They are the confrontation stage, the opening stage, the argumentation stage, and the concluding stage, each of which consists of various argumentative moves aimed at settling the disagreement in a reasonable manner [24]. In the confrontation stage, a difference of opinion is explicitly or implicitly established when one party’s standpoint encounters doubts or objections from the other party. In the opening stage, the parties try to resolve the difference of opinion by agreeing on the division of the burden of proof (protagonist vs antagonist) and the point of departure which features shared knowledge, values and discussion rules, etc. In the argumentation stage, the protagonist defends his/her standpoint by presenting a series of arguments which form simple or complicated argumentation structures. When the arguments are connected with the standpoint based on various logical relations, argument schemes are formed, the analysis of which is helpful to evaluating the soundness of the arguments advanced to defend the standpoint. In the concluding stage, the argumentative discussion comes to an end and the parties assess whether the difference of opinion has been resolved or to what extent it has been resolved.

Real-life argumentative discussions, however, do not necessarily unfold in the sequence of the four stages presented above. As van Eemeren observed, sometimes, in the course of a discussion, “the difference of opinion has been decided in its favor before the argumentation stage has even been completed”, and sometimes, “the parties realize they have failed to clearly identify what exactly they disagree on, so that it becomes necessary to go back to the confrontation stage” [24, p.22]. Therefore, it is essential to identify the four stages from the seemingly unstructured discourse and clarify the potential roles they play in the resolution process with such analytical instruments as differences of opinion, typologies of standpoints, argumentation structures and argument schemes, etc.

The second step is to clarify the “dialectical route” which is composed of a series of argumentative moves identified in the analytic overview and presents the argumentation pattern involved in the argumentative process. An argumentation pattern is, to a large extent, shaped by “a particular argument scheme or combination of argument schemes used in a particular kind of argumentation structure”[22, p. 15].

The primary argument schemes distinguished in pragma-dialectics are based on symptomatic, comparison, and causal relations, each of which corresponds to a type of argumentation[22]. In the symptomatic argumentation, the standpoint is defended by illustrating in the argument a specific sign, symptom, or prominent feature of what is stated in the standpoint [24]. For example, in the statement “John is a diligent student, because he always submits his assignments on time”, the argument (John always submits his assignments on time) is cited as a sign or symptom of what is claimed in the standpoint (John is a diligent student), which results in a symptomatic scheme.

In the comparison argumentation, the standpoint is defended by presenting the resemblance between what is claimed in the standpoint and what is stated in the argument [24]. This type of argumentation is common in homework tutoring contexts when a parent tries to motivate the child to overcome difficulties by comparing the child’s success in the past with the possibility of success at present. When a mother says “You’ll learn pinyin well (standpoint), because you learned all the English words quite well (argument)”, she defends the standpoint by implying the similarities between pinyin learning and English words learning on the grounds that both learning activities involve spelling and are time-consuming. Since the child succeeded in English vocabulary learning, he/she would overcome difficulties in pinyin learning.

In the case of causal argumentation, the standpoint is defended by showing a causal connection between the standpoint and the argument, whether the connection is of a cause to an effect, or a means to an end, or an action with a result [24]. It’s noteworthy that two sub-types of causal argumentation have been identified in pragma-dialectics as especially effective in justifying a standpoint. The first is “pragmatic argumentation” where the standpoint recommends a certain course of action and the argument points out a positive or negative result the action may cause [24]. The logical relation can be exemplified by the statement “Parents should establish a consistent bedtime routine for their children (standpoint), because it helps improve their sleep quality and overall health (argument)”, in which the standpoint that suggests a course of action is supported by the argument that indicates the favorable consequence. The second sub-type of causal argumentation is “complex pragmatic argumentation” which emphasizes the necessity of a solution asserted in the standpoint by presenting in the argument the ubiquity and severity of a problem [25]. For example, when a mother says, “You need to go to bed earlier, because your maths teacher has complained many times that you dozed off in class”, she supports her standpoint of a solution (to go to bed earlier) by stating the severity of a problem (the child dozed off in class). This solution-problem connection between the standpoint and the argument gives shape to the complex pragmatic argumentation.

While argument schemes presents the ways a standpoint is linked to the arguments, the argumentation structure displays the organization and complexity of argumentation. There are simple argumentation that only consists of a single argument, and multiple argumentation that contains a constellation of arguments and can be categorized into multiple, coordinative and subordinative argumentation [24].

In multiple argumentation, several arguments that are independent of each other and of equal weight are used to defend the same standpoint alternatively. In coordinative argumentation, a combination of arguments are employed to defend the standpoint jointly, as no single argument in the combination is sufficient to conclusively support the standpoint and the arguments have to complement each other. In subordinative argumentation, the defense of the standpoint is organized in layers, where the primary argument is supported by a sub-argument which may itself be reinforced by further sub-subarguments until the defense appears conclusive.

Building on the two steps discussed above, the final stage of argumentative discourse analysis involves tracking down the strategic considerations underlying the discourse. Strategic considerations reveal the “modes of strategic maneuvering and argumentative strategies” employed by arguers to achieve rationality and effectiveness in argumentation, largely influenced by the “institutional preconditions of the macro-context in which the argumentative discourse takes place” [23, p.13]. The instruments for the analysis of strategic considerations include those elements critical to the four discussion stages: standpoint, point of departure, argument schemes, etc. Among them, the most prominent characteristics pertaining to strategic maneuvering should be elaborated on to lay the groundwork for the identification of the argumentative style.

### 2.2. Categories of argumentative styles

The overall analysis of an argumentative discourse sets the stage for identifying the argumentative style. By drawing on the key characteristics of different argumentative styles outlined in pragma-dialectics, the argumentative style of an discourse can ultimately be determined. Based on the different features embedded in the three dimensions of strategic maneuvering, two basic categories of argumentative styles have been identified: the detached argumentative style and the engaged argumentative style [23,24,26].

When an argumentative discourse features a detached argumentative style, its strategic maneuvering shows a consistent focus on rationality, objectivity, and impartiality in the three dimensions throughout the four discussion stages. More specifically, the detached argumentative style emphasizes “radiating objectivity” in the dimension of topical selection, conveys “reliability” in the dimension of adaptation to audience demand, and expresses “openness to an independent judgment” in the dimension of presentational devices [26, p. 513]. Those dimensional characteristics manifest themselves in more specific but consistent properties in different discussion stages, namely the confrontation stage, the opening stage, the argumentation stage and the concluding stage.

On the other hand, when an argumentative discourse features an engaged argumentative style, its strategic maneuvering highlights personal involvement, audience connection, and emotional appeals. The engaged argumentative style is characterized by “radiating commitment” in the dimension of topical selection, “communality” in the dimension of adaptation to audience demand and “inclusiveness” in the dimension of presentational devices [26, p. 513]. Those characteristics are also presented through more specific yet consistent properties across the different stages of an argumentative discussion.

The specific characteristics of the two argumentative styles are described in Table 1 [23, p. 16] and served as a major reference when we tried to discern the argumentative style of the sample conversation.

**Table 1:** Characteristics of detached and engaged argumentative styles in the four stages of argumentative process.

	Detached argumentative styles	Engaged argumentative styles
	**Confrontational argumentative style**	
**Topical selection**	Businesslike selection of what is to be discussed	Selection of issues showing the arguer’s involvement in the case
**Adaptation to audience demand**	Ensuring preservation of intersubjectivity	Connecting with presumed interests of the audience
**Choice of presentational devices**	Expressing independence	Expressing personal involvement
	**Opening argumentative style**	
**Topical selection**	Starting points consisting primarily of verifiable facts and generally recognized norms	Starting points demonstrating the arguer’s association with the cause
**Adaptation to audience demand**	Starting points that are likely to be considered indisputable by the audience	Starting points showing the arguer’s identification with what is important to the audience
**Choice of presentational devices**	Expressing a focus on a straightforward presentation with references to relevant data and rules	Expressing a focus on inclusiveness
	**Argumentational argumentative style**	
**Topical selection**	Arguments pointing out concrete results, advantages or another clear rationale for accepting the standpoint	Arguments putting the standpoint in a familiar light or making it easier to judge its acceptability
**Adaptation to audience demand**	Arguing in a way that makes the audience consider the rationality of accepting the standpoint	Arguing in a way that connects the standpoint at issue with the frame of reference and preferences of the audience
**Choice of presentational devices**	Expressing level-headedness and impartiality	Expressing empathy and compassion
	**Concluding argumentative style**	
**Topical selection**	Making clear which conclusion is made plausible by the argumentation that has been advanced	Embracing the conclusion that is reached emphatically as the favored outcome of the argumentative process
**Adaptation to audience demand**	Making the audience realize that the conclusion is the rational consequence of the argumentative process	Making the audience realize that the conclusion is based on the argumentative process the parties have gone through together
**Choice of presentational devices**	Presenting the conclusion that is reached matter-of-factly in a reporting manner	Presenting the conclusion that is reached in an appealing way to the audience

## 3. Method

### 3.1. Research design

This study is centered around a case analysis to investigate the argumentative style of parent-child interaction in the Chinese cultural context. By examining a single case from a corpus of parent-child conversations, it provides an in-depth analysis of argumentative strategies, reasoning patterns, and cultural influences involved in real-life family communication. The case study approach integrates theoretical insights from pragma-dialectics with empirical data to uncover culturally specific argumentative practices adopted by the parent and their implications for family education.

The case study follows a structured analytical process grounded in the pragma-dialectical framework as outlined in Section 2. The analytical instruments such as differences of opinion, typologies of standpoints, argumentation structures and argument schemes are utilized to reconstruct argumentative discourse, evaluate argumentative strategies and identify styles. These pragma-dialectic instruments enable a structured examination of how arguments are developed, defended, and refuted within the discourse, providing a systematic method for understanding the dynamics of argumentation.

The analytical process begins with an argumentative discourse analysis to clarify the analytic overview, dialectical routes and strategic considerations involved in the selected conversation. It then proceeds to identify the argumentative styles of the different stages reconstructed from the conversation. Based on the two steps, the predominant argumentative style is to be determined and its implications will be explored.

### 3.2. Data collection

This study is part of a project that focuses on the parent-child interactions in Chinese families. The project was carried out according to the guidelines of the Declaration of Helsinki and approved by the IRB of Zhejiang Gongshang University Hangzhou College of Commerce. The process of participant recruitment lasted about a year (from Sept 10, 2022 to Sept 30, 2023). The participants are personal acquaintances of the research team, including parents in their late 30s and their children of elementary school age (7-12 years old). The parents’ written consent for their own and their children’s participation was obtained. About 20 hours of recordings of parent-child conversations concerning educational topics from 5 Chinese families were collected (the length of each recording varies from 5 to 20 minutes). The conversations were recorded by the participants themselves in various settings such as meal time, the ride to school or home, field trips and homework tutoring, etc. The conversation chosen for the present study took place between a mother and a son during homework tutoring.

### 3.3. Transcription procedures

The recording analyzed in the study was first transcribed by an automatic speech recognition app and the transcript was subsequently revised and translated by the two authors. The coding adhered to the standards of Computerized Language Analysis (CLAN) from the Child Language Data Exchange System (CHILDES). CLAN supports the transcription and coding of language data in the CHAT (Codes for the Human Analysis of Transcripts) format that is widely used for creating consistent and detailed transcripts of spoken language [27]. The coded transcripts not only present the flow of a natural conversation by marking turn-taking features, but also include significant paralinguistic elements such as laughter, sighs, murmurs, etc., facilitating the identification of argumentative moves relevant to the resolution of differences of opinion in real-life interactions and the contextual factors that influence the effectiveness of argumentation. As such, this transcription method has been widely applied in parent-child argumentation research [2,6–8,10,11], contributing to reliable analysis of research data. The final version of the transcript for this study is presented below (the name of the child has been fictionalized for the sake of anonymity):

Participants: Mother (36 years old), Liuliu (7 years old, a second grader in elementary school)

%act: The mother is dictating sentences to Liuliu. Incorrect words are required to be copied five times and then the sentences will be dictated again.

1. → *Mother: Shouldn’t you know how to write “勃” in “王勃”((the name of an ancient Chinese poet))?
2.  → *Liulu: No, the teacher hasn’t taught it.
3.  → *  Mother: A “十”, a “冖”, a “子”, and a “力”.
*Mother: Is the word “坠”((fall)) correct? Take a good look at it by yourself! It consists of “阝”, “人” and “土”. What did you write here? It’s neither “坚”((firm)) nor “坠”.
*Mom: Don’t mix up the two words. Both contain the part of “土”. The one that consists of “队” and “土” is “坠”, okay?
*Liuliu: Okay.
*Mom: Then the one with “II”, “又” and “土” is “坚”. It’s the “坚” in “坚硬”((firm and hard)) and “益坚” ((firmer)).
*Liuliu: How does the “坚” in “益坚” end up wrong?
*Mom: Yeah, you have a lot to copy. How many incorrect words are there in total?
*Liuliu: “葛”((a surname)) is missing from the first sentence.
*Mom: Where is the period, honey?
*Liuliu: [=！Laughing]
*Mom: [=！Mimicking Liuliu’s laughter]
*Mom: “坚”, “坠”, “葛” in “诸葛亮”((the name of a celebrated statesman in ancient China)) and “勃” in “王勃”.
*Liuliu: One, two, three, four.
*Mom: You need to copy the four words five times. Here we go. Just write them here.
*Liuliu: Four times five equals twenty!
*Mom: Okay, hurry up. Let’s do the dictation again in a while.
*Liuliu: Do the dictation again?
*Mom: It’s very quick, right? Otherwise, what are you going to do tomorrow? Do you want to give a good performance in tomorrow’s dictation or not? Do you want to copy the words again?

%act: Liuliu starts copying the incorrect words.

→ *Mom: The “葛” in “诸葛亮”. ((Liuliu forgot to copy the word “葛”))
18. *Liuliu: If I write it wrong tomorrow, I have to copy this sentence six times.
19. *Mom: Yes, it’s better to get it right now, right?
20. *Liuliu: I’m already copying. [=！Murmuring softly]
21. *Mom: Do I make sense?
22. *Liuliu: Yes.
23. *Mom: Okay.
24. *Liuliu: Let’s do the dictation again later.
25. *Mom: Okay.
26. *Liuliu: I’ve copied them three times. Two more times.
27. *Mom: Well, in the end you still have to do five times. It’s fate, right?
→ *Mom: You can’t even write the “益”((one of the characters in the mother’s given name)) correctly. It’s in my name. You break my heart. ((Mother finds another incorrect word))
28. *Liuliu: [=！Laughing]
29. *Mom: You mistook “六” for the upper part of “益”. You must feel like “六六六” ((a buzz term coined by Chinese netizens, which means “awesome”)) . [=！Laughing]
30. *Liuliu: [=！Laughing] Mum, earlier I wrote dad’s name “朱波” as “朱泼”. [=！Laughing]
31. *Mom: 朱坡?
32. *Liuliu: Yes.
33. *Mom: The “坡” with “土”?
34. *Liuliu: No, the “泼”with “氵”.
35. *Mom: It should be “氵” plus “皮”.
36. *Liuliu: Yeah.
37. *Mom: You wrote “发” instead?
38. *Liuliu: Yes, look. [=！Laughing]
39. *Mom: [=！Laughing] You’re really funny.

%act: Liuliu continues to copy the incorrect words.

40. *Liuliu: I’ll copy them here.
*Liuliu: Mom, but I really don’t want to do the dictation again.
41. *Mom: You do better this time, because the result will be handed in to your teacher. It’s your homework.
42. *Liuliu: But twice!
43. *Mom: You made so many mistakes the first time around. You’re not familiar with those words.
*Mom: The left part of “波” is too big.
44. *Liuliu: Okay, then I’ll make it tiny.
45. *Mom: It’ll never be tiny.
46. *Liuliu: How about this time?
47. *Mom: Don’t you think it’s ugly? Look for yourself. You should take handwriting seriously.
*Mom: Alright, then, turn over to the next page and let’s do the dictation again.
48. *Liuliu: If there’s another mistake, do I have to copy it again?
49. *Mom: Believe in yourself. I think you’ll get the sentences all correct, won’t you?
50. *Liuliu: Yeah, all the words in the first sentence are correct.
51. *Mom: Yes. Don’t forget the period.

Note. %act: the actions of the speakers; * : a turn; → : extension of a turn;

(()): explanation of the adjacent speech; [=!]: paralinguistic features

### 3.4. Data analysis

To identify the argumentative style of the parent-child interaction, we first carried out an argumentative discourse analysis to clarify the analytic overview, dialectical route and strategic considerations, as explained in the analytical framework above.

#### 3.4.1. The analytic overview.

To identify the argumentation style of the above parent-child conversation, the discourse must first be reconstructed to determine the argumentative moves related to the resolution of the difference of opinion in the four stages. Therefore, such analytical instruments as differences of opinion, typologies of standpoints, argumentation structures and argument schemes, etc. need to be employed.

a. The confrontation stage: the difference of opinion and the standpoint.

According to the conversation, the mother first asks Liuliu to copy the incorrect words from the first dictation five times, then requires him to do the dictation again, and finally expresses her faith that Liuliu would be able to write all the sentences correctly. The standpoint is presented retrogressively, i.e., “The sentences must be written correctly.” However, during the argumentative process, Liuliu expresses his dissatisfaction by questioning “Four times five equals twenty!” and “Do the dictation again?” He subsequently raises a clear opposition by saying “but I really don’t want to do the dictation again”. Evidently, there is a single difference of opinion mixed with one side stating objection to the other.

b. The opening stage: the point of departure.

The point of departure, as a set of facts, value judgments or universal viewpoints that both sides of the argumentation can accept, plays a critical role in resolving the difference of opinion. In the conversation, the mother requires Liuliu to do better in the second dictation and explicitly states “the result will be handed in to your teacher” and “it’s your homework”, resorting to authority and recognized norms as starting points which form an indisputable point of departure, i.e., the dictation result will be submitted as homework to the teacher.

c. The argumentation stage: the argumentation structure and argument schemes

To persuade Liuliu to accept her standpoint, the mother employs a series of arguments which are reconstructed in the following argumentation structure:

Standpoint: The sentences must be written correctly.

Argument 1: Copy incorrect words five times.
1.1a Shouldn’t you know how to write “勃” in “王勃”? (“勃” is written incorrectly)
1.1a.1a A “十”, a “冖”, a “子”, and a “力”.
1.1b What did you write here? It’s neither “坚” nor “坠”. (“坚” and “坠” are mixed up)
1.1b.1a The one that consists of “队” and “土” is “坠”
1.1b.1b Then the one with “II”, “又” and “土” is “坚”
1.1c The “葛” in “诸葛亮”. (“葛” is missing)
1.1d You can’t even write the “益” correctly. It’s in my name. You break my heart. (“益” is written incorrectly)
1.1d.1a You mistook “六” for the upper part of “益”. You must feel like “六六六”.
1.1e (“波” and “泼” are mixed up)
1.1e.1a It should be “氵” plus “皮”.
1.1e.1b You wrote “发” instead? You’re really funny.
1.1e.1c Don’t you think it’s ugly? You should take handwriting seriously.
Argument 2: Let’s do the dictation again in a while.
2.1a It’s very quick.
2.1b Do you want to give a good performance in tomorrow’s dictation or not? Do you want to copy the words again?
2.1c You do better this time, because the result will be handed in to your teacher. It’s your homework.
2.1d You’re not familiar with those words.
2.1d.1a You made so many mistakes the first time around.
The argument schemes

According to the first level of the argumentation structure, *copying incorrect words five times* (Argument 1) and *doing dictation again in a while* (Argument 2) are effective means to ensure that *the sentences are written correctly* (standpoint), thus reasoning from *Argument 1* and *Argument 2* to the standpoint forms the general type of causal argumentation. At the second level, the mother’s proposal to copy incorrect words is based on the many errors in Liuliu’s first dictation, including all the examples from *1.1a* to *1.1e*. The reasoning pattern that argues for the necessity of a solution by presenting the ubiquity and severity of a problem constitutes complex pragmatic argumentation which is a sub-type of causal argumentation. Furthermore, *2.1a* emphasizes the swift and convenient features of the dictation process, forming symptomatic argumentation with *Argument 2*. Besides, *2.1b* and *2.1c* discuss the favorable outcomes of *doing dictation again*, forming pragmatic argumentation with *Argument 2,* which is also a sub-type of causal argumentation. Similar to the examples mentioned in *1.1a-1.1e*, *2.1d* stresses the problem Liuliu has with the words and forms complex pragmatic argumentation with *Argument 2*. At the third level, the mother employs symptomatic argumentation to identify those important components of the words so that Liuliu could better understand the differences between certain words.

d. The concluding stage: the outcome

Toward the end of the conversation, the mother expresses her faith in Liuliu and through Liuliu’s affirmative response it can be seen that they finally reach an agreement. Therefore, the difference of opinion was eventually resolved.

#### 3.4.2. The dialectical route.

**Fig 1:**
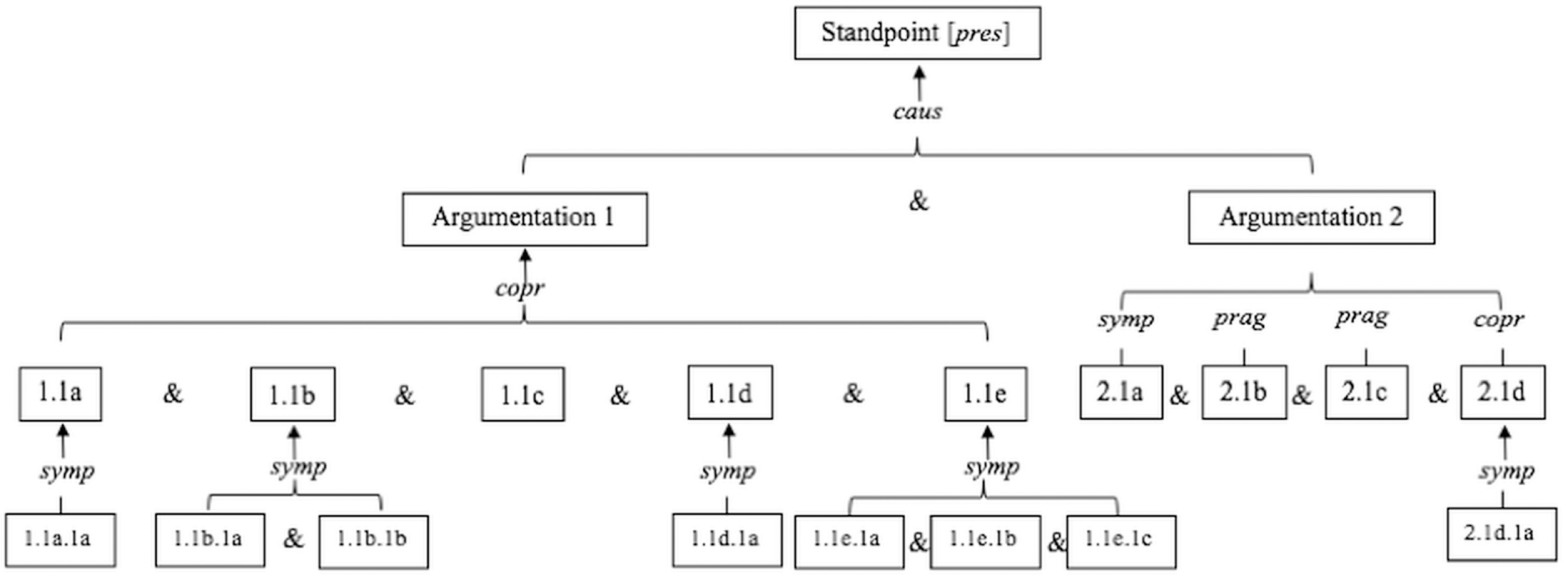
Dialectical route of the parent-child interaction. pres: prescriptive standpoint; caus: causal argumentation; copr: complex pragmatic argumentation; prag:pragmatic argumentation; symp: symptomatic argumentation; &: coordinative argumentation.

The presentation of the dialectical route draws on the argumentative moves identified in the analytic overview, in particular the argument schemes and argumentation structure that constitute the argumentative patterns. As shown in Fig 1, the parent-child conversation unfolds around a prescriptive standpoint, featuring the coordinative argumentation structure and various types of argumentation based on various argument schemes, such as symptomatic and causal argumentation, and their sub-types. The dialectical route shows to a certain extent the complexity and multidimensional nature of the argumentative discourse.

#### 3.4.3. The strategic considerations.

The strategic considerations of the parent-child interaction are characterized by two types of argumentation the mother adopts to convince her son. One is complex pragmatic argumentation to highlight the problems Liuliu has in handwriting and justify the necessity of *copying incorrect words five times*. The other is symptomatic argumentation to explain the essential components of the Chinese characters, which can effectively help Liuliu correct wrong words. Both types of argumentation are tailored for the audience by strategically aligning the reasoning with the educational context, because complex pragmatic argumentation emphasizes the necessity of repetitive practice to ensure long-term handwriting accuracy, while symptomatic argumentation helps the audience understand the essential components of the Chinese characters so that he can effectively correct mistakes and improve his handwriting.

The use of those argumentative strategies offers a glimpse into the argumentative style of the parent-child interaction, in which the arguments put the standpoint in a familiar light to the audience and make it easier to judge its acceptability.

## 4. Results

Based on the above analysis of the argumentative discourse and the characteristics of detached and engaged argumentative styles summarized in Table 1, this section presents the properties of the argumentative moves in the confrontational stage, opening stage, argumentation stage and concluding stage so as to identify the predominant argumentative style of the parent-child interaction.

### 4.1. Characteristics of the confrontational style: engaged

In the confrontational stage, the difference of opinion and the standpoint of the mother are not explicitly stated. Through the analytic overview, however, it can be learned that the mother puts forward two requirements: *copy the incorrect words five times* (Argument 1) and *do the dictation again in a while* (Argument 2). Based on the two arguments as well as the mother’s concluding words of “I think you’ll get the sentences all correct”, the mother’s standpoint is presented retrogressively as: *The sentences must be written correctly*. It is evident that the mother does not make a business-like assertion of her standpoint at the very beginning, but gradually derives the rationality of the standpoint through a series of arguments, manifesting an engaged style in topical selection.

Although the mother proposes a prescriptive standpoint which seems rather dominant and firm, her purpose is to ensure that Liuliu could perform well in the dictation at school the next day, which can be inferred from *2.1b* (*Do you want to give a good performance in tomorrow’s dictation or not? Do you want to copy the words again?).* Out of concern and care for Liuliu’s interest, the arguer’s adaptation to audience demand at this stage also characterizes an engaged style.

Finally, the standpoint is not directly expressed in the confrontational stage, but gradually presented as the conclusion at the end of the conversation. In *Turn 49* (“I think you’ll get the sentences all correct, won’t you?”), the mother uses a rhetorical question to imply that the ultimate goal of the assignment is enabling Liuliu to write the sentences all correctly. This presentational device does not highlight the arguer’s intention of seeking truth from facts but incorporates her subjective judgment and encouragement, thus exhibiting features of an engaged style. This result combined with our findings in topical selection and adaptation to audience demand suggests the arguer’s utilization of an engaged argumentative style in the confrontational state.

### 4.2. Characteristics of the opening style: detached

In the opening stage of an argumentative discourse, both sides usually agree on a point of departure. The analytic overview reveals that the point of departure of the parent-child interaction is indicated in *2.1c* (*You do better this time, because the result will be handed in to your teacher. It’s your homework.*), which highlights objective facts by resorting to authority and norms without the arguer’s passionate association with the cause, therefore exhibiting a somewhat detached style in topical selection.

For Liuliu, the emphasis on the nature of the dictation as homework to be handed in to the teacher makes the requirement more impersonal and mandatory. The point of departure is chosen as an established norm to address the objection of the audience without involving emotional appeals or showing compromise, resulting in detached characteristics in adaptation to audience demand.

In terms of the way the point of departure is expressed, the mother straightforwardly proposes her requirement (“you do better this time”), emphasizes the importance of dictation by appealing to authority (“because the result will be handed in to your teacher”) and explicitly states the practical goal of dictation (“it’s your homework”). Apparently, the presentation of the point of departure reflects a sense of rules and authority with little inclusiveness and involvement, characterizing a detached style. Therefore, the argumentative style of the opening stage, quite opposite to that of the confrontational stage, is predominantly detached.

### 4.3. Characteristics of the argumentational style: engaged

In the argumentation stage, the mother presents two arguments to defend her standpoint, i.e., Liuliu needs to *copy the incorrect words five times* (Argument 1) and *do the dictation again in a while* (Argument 2). *Argument 1* is justified by three types of errors in Liuliu’s handwriting: incorrectly written words, missing words, and confused words. These errors are proofs that the dictation result is full of problems which need to be addressed in a certain way. The complex pragma-dialectical argumentation utilized here is aimed at making Liuliu aware of the urgency to solve the current problems. The mother then used the symptomatic argumentation to explain the components of the incorrect words by naming the various radicals, which is in line with the learning habit and mindset of a second grader. *Argument 2* is first defended by the fact that dictation is a very swift process (2.1a) and then by the favorable outcomes (2.1b, 2.1c) which are followed by the problems Liuliu has with the words (2.1d). The symptomatic and pragmatic argumentation used in this part is aimed at motivating Liuliu to accept the dictation requirement. In this stage, the mother emphasizes the urgency of solving the problems and defends the standpoint in a way that is familiar to and acceptable by the audience, thereby adopting an engaged rather than a detached style in the dimension of topical selection.

As discussed in topical selection, the mother fully considers Liuliu’s cognitive level and learning habits when defending the standpoint by utilizing argument schemes that would be easy for Liuliu to accept. It can be seen that the arguer attempts to connect the standpoint with the audience’s frame of reference, presenting an engaged style in terms of adaptation to audience demand.

The exploitation of presentational devices in this stage is characterized by more personalized and vibrant expressions. First of all, the mother used a series of rhetorical questions to pinpoint Liuliu’s problems, such as “Shouldn’t you know how to write ‘勃’ in ‘王勃’?”, “Don’t you think it’s ugly?”, “Do you want to give a good performance in tomorrow’s dictation or not? Do you want to copy the words again?”. Compared with rational and neutral declarative statements, these questions are tinged with personal emotions. Meanwhile, the mother uses humorous and ironic expressions to ease the tension. For instance, when pointing out the error in the character “益”, the mother associates it with her own name, complaining that Liuliu breaks her heart. Afterwards, she ironically compares the wrong part to the popular internet slang term “六六六” to achieve humorous effects. Similarly, when discussing the confusion between “波” and “泼”, the mother adopts an ironic tone by saying “you are really funny”. These ironic comments serve as “an argumentative strategy adopted by parents to persuade the children to withdraw or decrease the strength of their standpoint” [3, p.1389]. The exploitation of presentational devices in this stage shows the arguer’s empathy with the audience by means of detailed explanations and personalized expressions, indicating an engaged style. On the whole, the engaged characteristics of this stage are consistent in the three dimensions of the argumentative style.

### 4.4. Characteristics of the concluding style: engaged

In the concluding stage, the mother expresses her faith in Liuliu and retrogressively presents her standpoint that *the sentences must be written correctly*. The conclusion is emphatically embraced as the favored outcome of the argumentative process. Hence, the topical choice of the concluding stage is characterized by an engaged style.

After tutoring Liuliu in detail to correct the words and asking him to complete the copying task, the mother urges him to believe in himself and expresses her confidence in him. Liuliu’s affirmative response is evidence that the arguer makes the audience realize that the conclusion is based on the argumentative process they have gone through together. Therefore, adaptation to audience demand in this stage features an engaged style.

In terms of presentational devices, the mother used an imperative statement (“Believe in yourself.”) to boost Liuliu’s confidence and a rhetorical question (“I think you’ll get the sentences all correct, won’t you?”) to stress her trust in him. Instead of presenting the conclusion in a reporting manner, the arguer attempts to convince the audience in an appealing way which is indicative of an engaged style. On the whole, the concluding stage also demonstrates a consistent engaged style.

## 5. Discussion

The above analysis indicates that the overall argumentative style of the parent-child interaction can be qualified as engaged despite some detached features discerned in certain stage.

### 5.1. Predominant engaged features

The results suggest that the mother demonstrated her personal commitment in the argumentative process and reasoned with her child in a comprehensible and inclusive manner. These engaged argumentative features in family education context are in line with previous research on parental involvement in children’s education in China. Research by Huang and An[17], which surveyed 12,575 parents and children from 53 schools across China, identified four distinctive types of parental involvement in children’s education in Chinese households: supportive, permissive, restrictive, and neglectful, among which supportive and permissive involvement accounted for 74 percent of parental involvement (with the former taking up 20% and the latter 54%). The two types of involvement were characterized by such parental behaviors as supervising, checking, tutoring, assigning extra homework, and parents’ positive engagement in and commitment to children’s education. In our case, the mother’s involvement style clearly aligns with both supportive and permissive involvement, offering a relevant and illustrative example of effective parental engagement in the Chinese educational context.

Given the fact that the audience in this case is in the lower grade, the mother, as the homework tutor, reasoned and argued in a manner understandable and acceptable to the audience. Whether it was the proposition of the standpoint, the development of the argumentative process or the deduction of the conclusion, the mother avoided being overly neutral and objective in topical selection, adaptation to audience demand, and the exploitation of presentational devices by adopting effective argument schemes such as complex pragmatic argumentation to highlight the problems Liuliu had in handwriting and symptomatic argumentation to explain the essential components of the Chinese characters. The engaged argumentative style observed in the case is similar to the conclusion drawn by Bova [6] who pointed out that a typical feature of parent-child interactions was that parents usually use argumentative strategies and expressions easy for children to understand to persuade them and that this feature was determined by the communicative activity type to which parent-child argumentation belongs.

### 5.2. Occasional detached features

The detached argumentative style manifests itself in the opening stage through a seemingly indisputable point of departure. The mother resorted to the teacher as authority and stressed the nature of the assignment as homework, implying that the point of departure chosen here was recognized norms and rules the audience must follow. As Bova [5, p. 133] argued, “parents always refer to an adult as source of authority... such as a grandparent or a child’s teacher” and “children are more prone to accept their parents’ argumentation when the authority is another adult”. It suggests that certain impersonal and imposing arguments in parent-child interactions are somehow inevitable.

As a matter of fact, homework tutoring has become a significant part of family education in Chinese cultural context. Through homework tutoring, parents get to know their children’s academic performance and teachers manage to reinforce parents’ involvement in children’s education. In many regions of China, parents are requested by teachers to check each assignment on a homework list every day to ensure that their children have done the review and preview work appropriately. In Chinese family education settings, therefore, parent-child interactions tend to involve points of departures concerning teachers and school to ensure the successful proceeding of the argumentation.

### 5.3. Influence of cultural context on the argumentative style

The argumentative style observed in the parent-child interaction within this study is influenced by the cultural context, particularly the emphasis on education and academic success in Chinese families. The findings align with the broader understanding that in Chinese culture, education is highly valued and parents often see it as their responsibility to ensure their children’s academic achievements.

In our case, the mother’s engaged argumentative style exemplifies the high level of parental involvement in Chinese families where “parents not only oversee but actively participate in their children’s education” [19, p.62]. The mother’s use of effective argument schemes as well as personalized linguistic devices can be seen as a strategic choice that resonates with Chinese parents’ “constructive involvement” indicated by “positive emotions, autonomy support, and mastery-oriented teaching” [20, p.2] and beneficial for “children’s academic and emotional functioning” [20, p.9].

On the other hand, the occasional use of a detached style, particularly in the opening stage where the mother appealed to the authority of the teacher and the norms of homework, suggests a balancing gesture between nurturing involvement and instilling discipline and respect for authority figures. This mixed feature highlights the tension between being supportive while also enforcing rules and expectations in Chinese families— a dynamic that is likely influenced by the broader cultural context where the parent acts as a guide and an enforcer.

## 6. Conclusion

This study has provided an in-depth analysis of the argumentative style of parent-child argumentation within the context of homework tutoring in China, illustrating the predominant use of an engaged argumentative style by the parent, which is characterized by personal involvement, adaptation to the child’s needs, and the use of culturally resonant argumentation strategies. Occasionally, the parent employed a detached argumentative style, particularly in the opening stage where appeals to authority and adherence to norms were used to establish an objective and impersonal point of departure. This mixed property of argumentative styles reflects parental responsibility in ensuring children’s academic success in China and the broader cultural emphasis on educational norms.

In terms of methodological implications, the study has presented a case analysis that enables the exploration of the complexities and dynamics of a real-life parent-child interaction, which are difficult to capture through quantitative methods. This approach allows for the detailed observation of how argumentative strategies unfold in real time, providing insights into the subtle nuances of parent-child argumentation that might be overlooked in larger-scale studies.

Additionally, by resorting to the theoretical notion of Argumentative Style, the study has extended the analytical framework of parent-child argumentation by including not only the dialectical structure of argumentative discourse, but also the rhetorical properties of various argumentative moves, along with cultural and situational factors. This framework is instrumental in evaluating how parents resolve differences of opinion with their children by taking into account strategic maneuvering in diverse contexts.

While the case analysis offers insights into the analytical process of real-life parent-child argumentation, the study is limited by its focus on a single interaction within a specific family setting, which may constrain the generalizability of the findings. Additionally, the controlled settings and the reliance on self-recordings by the participants may have impacted the authenticity and reliability of the results. As such, it is recommended that future research involve a larger sample size and a more diverse range of interactions between parents and children to explore the variability and consistency of argumentative styles in different settings. To enhance the authenticity of the data, the process of data collection could involve a third party, such as another family member not directly involved as a participant, who manages to record the conversations. This approach may better capture genuine parent-child argumentative interactions in everyday contexts.

## Supporting information

S1 Recording The recording of the parent-child conversation. (MP3)

S2 Text The original transcript of the recording. (DOCX)

S3 Translated Text Translation of the transcript. (DOCX)
