# Supplementary material for: Argumentative style of parent-child interactions: A case study
Source: PLoS One. 2025 Mar 18;20(3):e0318310. doi: 10.1371/journal.pone.0318310 (PMC11918318; doi:10.1371/journal.pone.0318310)
Supplement: S2 Text — (DOCX) [file pone.0318310.s002.docx]

抄一下，加深一下印象，意思也要抄五遍。嗯王勃的勃不是应该，应该应该应该会写吗？不是老师没教过。哦句号句号千万不要忘记掉。我补上了。嗯，不坠的坠你是不是写错了？你这看清楚。一个耳朵一个人一个土，你这写的是是啥呀。坚不像坚，坠不像坠啊，你这两个字不要搞错了，两个都是土字底的。一个队伍的队，一个土是坠知道吗？然后一个一个一个两竖一个又一个土是坚，坚硬的坚，益坚的坚。怎么益坚的坚也写错啊，你要抄的很多呢。几个字啊一共。这第一句话你的句号去哪里？亲。呵呵呵呵，坚， 坠，然后呢诸葛亮的葛。然后王勃的勃。两个三个四个。四个抄五遍，那你开始抄吧。就抄在这里。四五二十。嗯，快点。等一下再默一遍。还要默。默一下不是很快的吗？那你不然的话你明天怎么默呢。明天到时候你想明天默全对呢，还是说想想明天默错再重新抄呢。默错这句话要抄六遍。对呀那你总是现在默全对好的呀。对不对？我是不是我说的有没有道理？有。等会再默一遍。好的。这里抄两遍我就有吗？啊。那我再在这里抄两遍。你最终还是得超五遍。命运决定了你要抄五遍对不对？你连施益红的益都不会写，你太让我伤心了。还写个六了。你说自己666还是我。你会写朱吗？我会写啊。朱我怎么不会写。我把爸爸姓名朱波写成了朱波。朱什么。我写土字旁那个坡啊。爸爸朱三点水的那个泼。他三点水的波呀，三点水加个皮，你三点水加了个拔的半边那个是吧。你写的发是吧。啊就是你看，呵呵呵。你真搞笑。我抄这。还一个王勃的勃。你怎么这都会忘记呢？自己会不会写字，都不会，都忘记了。妈妈，但是我真的不想再默一遍。你下一遍写好点要教去给老师的啦肯定，是不是。但是两遍啊。那你第一遍错那么多呀。说明你还不会啊这些字。一个十一个秃宝盖一个子一个力。葛亮。这波左边写太大了啊。嗯，那我就写迷你哦。你再迷你也迷你不到哪里去。你说难看不难看，自己自己看看，难看不难看。写字不是开玩笑的。好了，嗯，翻页再默一遍。如果还有错还要抄。你要相信你自己。你肯定是全对的。我觉得对吧。第一句话就全对的。对呀，句号不要忘记了, 再提醒你一遍，忘记了又要抄啊。嗯专门抄句号抄100遍。哈哈哈。因为你句号经常掉的，你有没有发现？考试的时候也掉句号。我就掉过一次。会止一次吗？我等下把你试卷翻出来看一下。那一年级的时候不算？不要写那么难看，要交的。第一遍才交，这才交。
