# Supplementary material for: Argumentative style of parent-child interactions: A case study
Source: PLoS One. 2025 Mar 18;20(3):e0318310. doi: 10.1371/journal.pone.0318310 (PMC11918318; doi:10.1371/journal.pone.0318310)
Supplement: S3 — (DOCX) [file pone.0318310.s003.docx]

Copy this down to reinforce your memory. You mean to copy it five times as well? Yes. Shouldn’t you know how to write “勃” in “王勃”? No, the teacher hasn’t taught it. A “十”, a “冖”, a “子”, and a “力”. I see. Don’t forget the period. I have added it. Is the word “坠”correct? Take a good look at it by yourself! It consists of “阝”, “人” and “土”. What did you write here? It’s neither “坚”nor “坠”. Don’t mix up the two words. Both contain the part of “土”. The one that consists of “队” and “土” is “坠”, okay? Okay. Then the one with “II”, “又” and “土” is “坚”. It’s the “坚” in “坚硬”and “益坚”. How does the “坚” in “益坚” end up wrong? Yeah, you have a lot to copy. How many incorrect words are there in total? “葛” is missing from the first sentence. Where is the period, honey? “坚”, “坠”, “葛” in “诸葛亮” and “勃” in “王勃”. One, two, three, four. You need to copy the four words five times. Here we go. Just write them here. Four times five equals twenty! Okay, hurry up. Let’s do the dictation again in a while. Do the dictation again? It’s very quick, right? Otherwise, what are you going to do tomorrow? Do you want to give a good performance in tomorrow’s dictation or not? Do you want to copy the words again? The “葛” in “诸葛亮”. If I write it wrong tomorrow, I have to copy this sentence six times. Yes, it’s better to get it right now, right? I’m already copying. Do I make sense? Yes. Okay. Let’s do the dictation again later. Okay. I’ve copied them three times. Two more times. Well, in the end you still have to do five times. It’s fate, right? You can’t even write the “益”correctly. It’s in my name. You break my heart. You mistook “六” for the upper part of “益”. You must feel like “六六六”. Mum, earlier I wrote dad’s name “朱波” as “朱泼”. “朱坡”? Yes. The “坡” with “土”? No, the “泼”with “氵”. It should be “氵” plus “皮”. Yeah. You wrote “发” instead? Yes, look. You’re really funny. I’ll copy them here. Mom, but I really don’t want to do the dictation again. You do better this time, because the result will be handed in to your teacher. It’s your homework. But twice! You made so many mistakes the first time around. You’re not familiar with those words. The left part of “波” is too big. Okay, then I’ll make it tiny. It’ll never be tiny. How about this time? Don’t you think it’s ugly? Look for yourself. You should take handwriting seriously. Alright, then, turn over to the next page and let’s do the dictation again. If there’s another mistake, do I have to copy it again? Believe in yourself. I think you’ll get the sentences all correct, won’t you? Yeah, all the words in the first sentence are correct. Yes. Don’t forget the period. I’ll remind you again. If you forget it, you’ll have to copy it again. Hmm, you’ll have to copy the period 100 times. Hah. Because you often forget the period, haven't you noticed? You forget the period even during exams. I only forgot once. Only once? Let’s check your test papers later. First grade doesn’t count. Don’t write so messily. It has to be handed in to the teacher. The first dictation result gets handed in. This is the one.
